# Quantifying Subresolution 3D Morphology of Bone with Clinical Computed Tomography

**DOI:** 10.1007/s10439-019-02374-2

**Published:** 2019-10-03

**Authors:** S. S. Karhula, M. A. J. Finnilä, S. J. O. Rytky, D. M. Cooper, J. Thevenot, M. Valkealahti, K. P. H. Pritzker, M. Haapea, A. Joukainen, P. Lehenkari, H. Kröger, R. K. Korhonen, H. J. Nieminen, S. Saarakkala

**Affiliations:** 1 Research Unit of Medical Imaging, Physics and Technology, University of Oulu, POB 5000, 90014 Oulu, Finland; 2 Infotech, University of Oulu, Oulu, Finland; 3 Medical Research Center, University of Oulu, Oulu, Finland; 4 Department of Anatomy Physiology and Pharmacology, University of Saskatchewan, Saskatoon, SK Canada; 5 Department of Surgery and Intensive Care, Oulu University Hospital, Oulu, Finland; 6 Department of Laboratory Medicine and Pathobiology, Surgery University of Toronto, Toronto, ON Canada; 7 Mount Sinai Hospital, Toronto, ON Canada; 8 Department of Diagnostic Radiology, Oulu University Hospital, Oulu, Finland; 9 Department of Orthopaedics, Traumatology and Hand Surgery, Kuopio University Hospital, Kuopio, Finland; 10 Department of Anatomy and Cell Biology, University of Oulu, Oulu, Finland; 11 Department of Applied Physics, University of Eastern Finland, Kuopio, Finland; 12 Department of Neuroscience and Biomedical Engineering, Aalto University, Espoo, Finland

**Keywords:** Grey-level co-occurrence matrix, Textural analysis, Micro-computed tomography, Cone beam computed tomography, Imaging, Osteoarthritis

## Abstract

The aim of this study was to quantify sub-resolution trabecular bone morphometrics, which are also related to osteoarthritis (OA), from clinical resolution cone beam computed tomography (CBCT). Samples (*n *= 53) were harvested from human tibiae (*N *= 4) and femora (*N *= 7). Grey-level co-occurrence matrix (GLCM) texture and histogram-based parameters were calculated from CBCT imaged trabecular bone data, and compared with the morphometric parameters quantified from micro-computed tomography. As a reference for OA severity, histological sections were subjected to OARSI histopathological grading. GLCM and histogram parameters were correlated to bone morphometrics and OARSI individually. Furthermore, a statistical model of combined GLCM/histogram parameters was generated to estimate the bone morphometrics. Several individual histogram and GLCM parameters had strong associations with various bone morphometrics (|*r*| > 0.7). The most prominent correlation was observed between the histogram mean and bone volume fraction (*r *= 0.907). The statistical model combining GLCM and histogram-parameters resulted in even better association with bone volume fraction determined from CBCT data (adjusted *R*^*2*^ change = 0.047). Histopathology showed mainly moderate associations with bone morphometrics (|*r*| > 0.4). In conclusion, we demonstrated that GLCM- and histogram-based parameters from CBCT imaged trabecular bone (*ex vivo*) are associated with sub-resolution morphometrics. Our results suggest that sub-resolution morphometrics can be estimated from clinical CBCT images, associations becoming even stronger when combining histogram and GLCM-based parameters.

**Electronic supplementary material:**

The online version of this article (10.1007/s10439-019-02374-2) contains supplementary material, which is available to authorized users.

## Introduction

Subchondral bone sclerosis, causing increases in bone volume fraction (BV/TV, ratio of bone volume and tissue volume), trabecular thickness (Tb.Th.), trabecular number (Tb.N.) and a decrease in trabecular separation (Tb.Sp.), has been associated with osteoarthritis (OA)-driven cartilage defects.^2^,^5^,^10^ These alterations in subchondral bone are often linked to the later stages of OA. However, in early OA, contrary structural alterations in subchondral bone have been reported within several animal models.^1^,^9^,^16^ Furthermore, an increase in subchondral bone resorption, due to abnormally high bone turnover, has also been observed to occur in progressive OA.^23^ The contradictory results in subchondral bone alterations in animal studies, and limited evidence of early OA-related subchondral bone alterations in human tissue, supports the need for further research on early OA-induced alterations of human subchondral bone at the micro- and nanostructural level.

Computed tomography (CT) modalities provide spatial resolution starting from 100 nm with synchrotron radiation nano-CT up to a 0.2–0.5 mm with clinical CT, enabling the hierarchical imaging of subchondral bone from the sub-cellular level to the organ level.^21^ Desktop micro-computed tomography (*µ*CT) imaging has become the gold standard for quantification of bone morphology and microstructure in 3D.^24^ When approaching to the spatial resolution of clinical CT, trabecular bone structure has been reported to be quantifiable up to 100 *µ*m voxel size.^17^ On the other hand, with dental CT-imaged bone, strong associations between the average of the grey-level values and microstructural trabecular bone morphometrics from *µ*CT have been reported in the literature.^15^

In order to quantify bone microarchitecture from clinical X-ray based imaging modalities, several texture analysis algorithms have been applied in osteoporosis and OA research applications. Notably, fractal analysis and run-length distribution texture analysis methods from X-ray radiographs, and variogram from dual-energy absorptiometry, have been reported to correlate with morphometric parameters of bone.^4^,^19^,^26^ Textural analysis of 2D radiographs has been shown to be sensitive to OA-related structural alterations and changes in bone density of subchondral bone.^13^,^14^,^19^ For volumetric data, grey-level co-occurrence matrix (GLCM) based texture analysis, first introduced by Haralick,^11^,^12^ has been previously applied to medical CT imaging of bone.^8^,^20^,^22^ Moreover, GLCM parameters have shown good correlations to the BV/TV and biomechanical properties of bone from *µ*CT data when the volumetric data is re-projected in 2D.^30^

GLCM-based textural analysis is a method to extract second order statistical features from grey-level images. In comparison to histogram based parameters (*i.e*. grey-level mean and standard deviation, and histogram skewness and kurtosis), GLCM is not related to the absolute value of the grey-levels but to the relationship of the pixel’s (or in 3D voxel’s) grey-level value to the grey-level values of its neighboring pixels/voxels. Thus, calculating GLCM and histogram-based parameters are complementary to each other. In the case of X-ray radiography and clinical CT imaging of subchondral bone, where resolution is insufficient for calculating the true morphometrics of bone, the grey-level values can still be reflective of the microarchitecture.

As CT modalities are used to study bone morphometrics at several hierarchical length scales, histogram and GLCM-based analysis of the grey-level values are likely to produce further information from the sub-resolution features. This will provide new tools to researchers, to help them analyze their data beyond the resolution limits of the CT system. This study aims to quantify sub-resolution bone morphometrics, which are related to OA, from clinical cone-beam CT (CBCT) *ex vivo*. We utilize GLCM texture and histogram-based parameters of the CBCT-imaged subchondral bone with various OA severities, and compare them with the morphometric parameters quantified from *µ*CT. We hypothesize that trabecular bone morphometrics (BV/TV, Tb.Th., Tb.N., etc.) quantified from *µ*CT associate with the GLCM parameters and histogram parameters calculated from the clinical CBCT data.

## Materials and Methods

**Figure 1:**
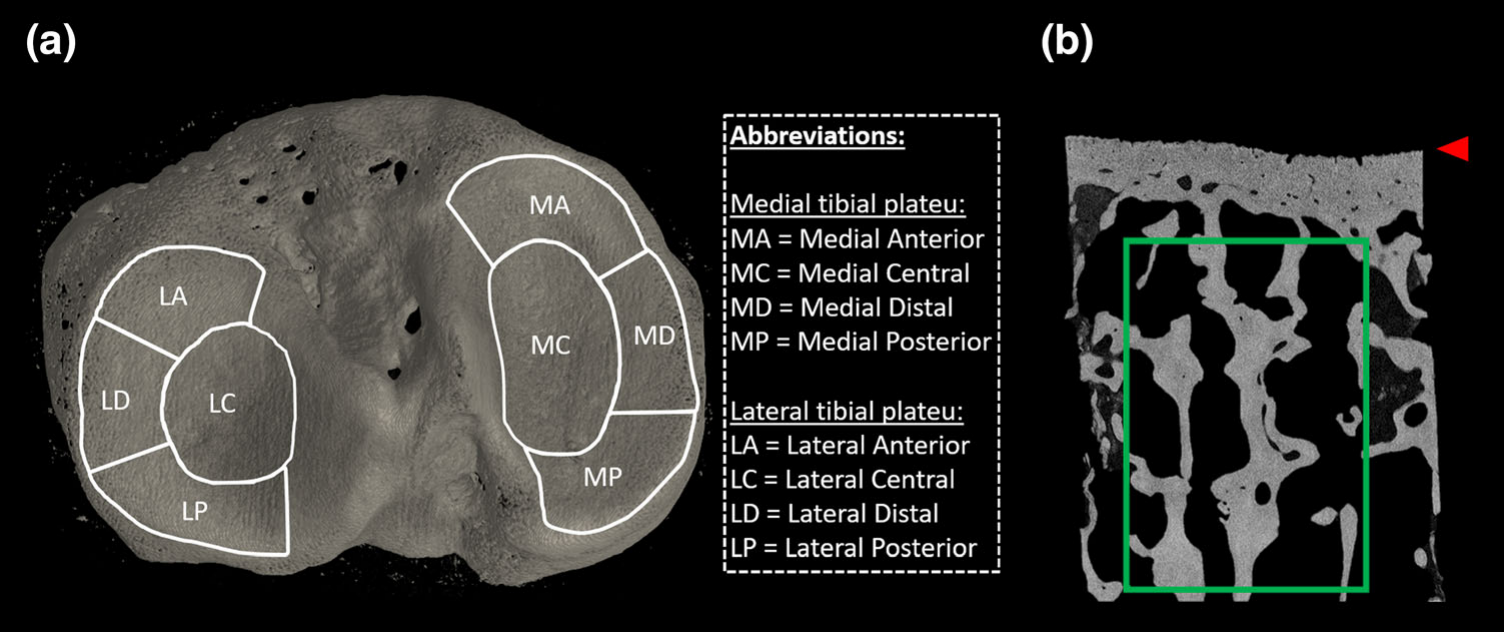
Core extraction and VOI selection. (a) 8 areas from tibial plateau from which the osteochondral cores were extracted. (b) Sagittal slice of *µ*CT imaged subchondral bone core on which the trabecular bone VOI (green rectangle) and calcified cartilage—articular cartilage interface (red arrow) are marked.

53 osteochondral cores (Ø = 4 mm) were extracted from total knee arthroplasty (TKA) patients and from cadavers without OA diagnosis under the approval of ethical committees of Northern Ostrobothnia Hospital District (Finland, Approval No. 78/2013) and Research Ethics Committee of Northern Savo Hospital District (Finland, Approval No. 58/2013 and 134/2015). Dental drill with a trephine blade was used for extracting the cores. From the 53 cores, 46 cores were extracted from the tibial plateaus of 2 TKA patients (females aged 68 and 73) and 2 cadavers (males aged 68 and 68). From cadavers, cores from both tibiae were included (see Table 1). Only one tibia per TKA patient was subjected to the core extraction, because sample harvesting from these patients is conducted from the remnant tibial pieces of the TKA operation. Cores were extracted from 8 manually determined locations on the tibial plateau (Fig. 1). The remaining 7 cores (out of 53) were extracted from the weight-bearing area of TKA patient femora (*N *=7, 5 females and 2 males, age range: 66–86) to ensure high variability in OA severity. After core extraction, samples were preserved in a − 80 °C freezer until thawed for imaging. Prior to imaging, samples were immersed in 4% saline buffered formaldehyde for fixation and imaged in the fixation media.

**Table 1:** Sub-groups of the osteochondral samples.

Grouping criteria	Number of cores	Patients/group
	*n*	*N*
Total number of cores	53	11 (9 TKA patients, 2 cadavers)
Sample origin		
TKA patients	15	2
Cadavers	38	2
Compartmental location^a^		
Medial tibial plateau	22	4 (2 TKA, 2 cadavers)
Lateral tibial plateau	24	4 (2 TKA, 2 cadavers)
Areal location^a^		
Central tibial plateau	10	4
Anterior tibial plateau	11	4
Posterior tibial plateau	12	4
Distal tibial plateau	13	4

Number of samples (*n*) used in parameter comparisons (total number of cores), and number of samples per subgroup for locational dependency analyses (Sample origin, Compartmental location, Areal location), and number of patients in each group are listed in the table.

^a^Femoral cores (*n* = 7, *N* = 7) excluded.

Harvested cores were imaged with desktop *µ*CT and with CBCT. The desktop *µ*CT imaging was conducted with a Skyscan 1272 (Bruker microCT, Kontich Belgium) with 50 kV, 200 *µ*A, 2.75 *µ*m voxel size, 2200 ms exposure time, 2 h 42 min scan time, 1200 projections from 360°, averaging 3 frames/projection, and with a 0.5 mm Al filter. Projection data were reconstructed with NRecon-software (v.1.6.10.4, Bruker microCT) with beam hardening and ring artefact corrections applied.

The CBCT imaging was conducted with the clinical extremity CBCT scanner (Planmed Verity, Planmed Inc., Helsinki, Finland) with 80 kV, 12 mA, 200 *µ*m voxel size, 2 min scan time, 300 projections and 20 ms pulse time. Reconstruction was conducted with the manufacturer’s own reconstruction software with “standard” reconstruction filter.

**Figure 2:**
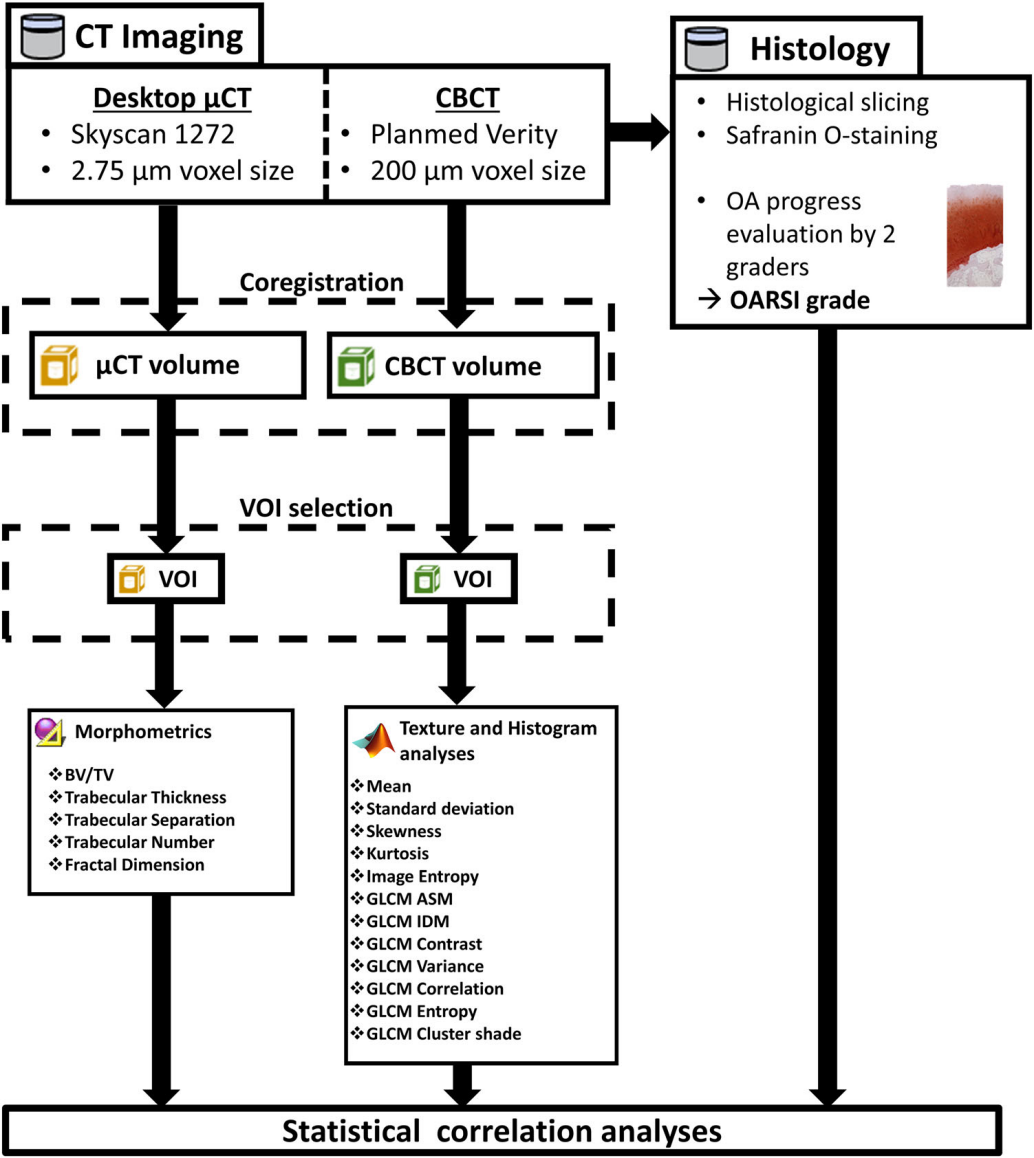
Flowchart describing the imaging and analysis methods used in this study.

CBCT and *µ*CT volumes were co-registered using rigid transformations with the Dataviewer-software (v.1.5.4, Bruker microCT). Each co-registered dataset was visually evaluated to confirm the successful co-registration. For the CBCT and *µ*CT comparison, cylindrical volume of interest (VOI) of 3000 × 3000 × Z *µ*m^3^ (where *Z* is the depth from the subchondral bone plate, range: 605–4435 *µ*m) was selected from the trabecular bone. Z was restricted based on how much trabecular bone was left in biopsies after the surgeries. The VOI was visually evaluated to be sufficient for each sample so that the minor alterations to the edges of trabecular bone core due drilling would be excluded from the analysis. The bone masks were utilized for the morphometric analyses of *µ*CT-imaged trabecular bone. Figure 2 presents the flowchart of the methods and analyses used.

CTAn software (v.1.17.7.2, Bruker microCT) was used to calculate the relevant 3D morphometrics of the bone from the *µ*CT data. We considered that the following trabecular bone morphometrics primarily affect to the grey-level values of CBCT imaged subchondral trabecular bone: BV/TV, Tb.N., Tb.Th., Tb.Sp., and local bone surface complexity (fractal dimension, FD). All the aforementioned morphometrics were calculated within the VOI of the *µ*CT-imaged trabecular bone data (Fig. 1).

The histogram and GLCM texture parameters were calculated from the CBCT data within the VOI described above. The histogram parameters (mean, standard deviation, skewness, kurtosis and image entropy) and GLCM texture parameters were calculated with Matlab (v.2017b, Mathworks, Natick, MA, USA).

To briefly summarize the calculation of the GLCM, the GLCM is a *N*_g _× *N*_g_ matrix where *N*_g_ is the number of quantized grey levels. The GLCM consists of elements *P*(*i,j*), representing the number of occurrences in grey-levels *i* and *j* within a certain window defined by the displacement *d* and angle *θ* for 2D images (and for 3D matrix [*θ*, *φ*]). The GLCM is then normalized by calculating the second order statistical probability values *p*(*i,j*) with Eq. 1:

1 $$p\left( {i,j} \right) = \frac{{P\left( {i,j} \right)}}{{\mathop \sum \nolimits_{i = 0}^{{N_{\rm{g}} - 1}} \mathop \sum \nolimits_{j = 0}^{{N_{\rm{g}} - 1}} P\left( {i,j} \right)}}.$$

In our study, 13 GLCMs from 13 individual angles of the 3D dataset (Supplementary Table 1) were calculated and summed to provide rotationally invariant texture features. The selection of parameters *d* and *N*_g_ affect to the calculated texture parameters.^7^ We selected the voxel displacement *d* to be 1, because in CBCT the morphometric features, to which we correlated with the texture parameters, were below our imaging resolution. For 8-bit grey-level images, the *N*_g_ can obtain values up to 256, reducing *N*_g_ from 256 can decrease the noise and calculation time of the analysis in the expense of diminution of the information. Previous studies^7^ have noted that the classification accuracy can be retained when *N*_g_ ≥ 24. In this study, *N*_g_ was selected to be 256 for the GLCM generation from the CBCT, to address the strong impact of the partial volume effect occurring in CBCT imaging. As the partial volume effect sums quantifiable micromorphometrics in relatively large voxels (as obtained from CBCT), it is necessary to keep *N*_g_ high to avoid additional loss of morphometric information, which occurs when grey-levels are grouped during the GLCM calculation.

From the available textural features, inverse difference moment (IDM), angular second moment (ASM), contrast, variance, entropy, and cluster shade were calculated from the summed GLCM. While several other parameters could be calculated from the GLCM, in this study, only parameters which have often been reported in literature were considered (Table 2).

**Table 2:** Description of GLCM textural parameters.

Textural feature	Equation^a^	Description
Contrast	$\mathop \sum \limits_{n = 0}^{{N_{\rm{g}} - 1}} \left( {i - j} \right)^{2} \left\{ {\mathop \sum \limits_{i = 1}^{{N_{\rm{g}} }} \mathop \sum \limits_{j = 1}^{{N_{\rm{g}} }} p\left( {i,j} \right)} \right\}$	Measure of local grey level variation in the image. The high values of the contrast can indicate the presence of large local gradient alteration in the image (e.g. edges, wrinkled textures)
Variance (sum of squares)	$\mathop \sum \limits_{i = 1}^{{N_{\rm{g}} }} \mathop \sum \limits_{j = 1}^{{N_{\rm{g}} }} \left( {i - {\mu }} \right)^{2} p\left( {i,j} \right)$	Describes global variance of the image. Variance puts high weights on grey-level values dispersing from the mean value of *p*(*i,j*). Unlike Contrast, Variance has no spatial frequency
Angular second moment (ASM, energy, uniformity)	$\mathop \sum \limits_{i = 1}^{{N_{\rm{g}} }} \mathop \sum \limits_{j = 1}^{{N_{\rm{g}} }} p\left( {i,j} \right)^{2}$	Describes the overall homogeneity of the image. In homogenous images, GLCM results in few high *p*(*i,j*) values which results in high sum of squares. Thus with increasing textural uniformity (or increasing homogeneity) the ASM will have increasing values
Inverse difference moment (IDM, homogeneity)	$\mathop \sum \limits_{i = 1}^{{N_{\rm{g}} }} \mathop \sum \limits_{j = 1}^{{N_{\rm{g}} }} \frac{{p\left( {i,j} \right)}}{{1 + \left( {i - j} \right)^{2} }}$	Measure of local homogeneity of an image. The weighting factor (1 + (*i *− *j*)^2^)^−1^ emphasizes the pixel pairs with close grey-scale values. This results in relatively higher IDM in homogenous images. IDM is often correlated to Contrast, as with high local homogeneity the Contrast tends to be low
Correlation	$\mathop \sum \limits_{i = 1}^{{N_{\rm{g}} }} \mathop \sum \limits_{j = 1}^{{N_{\rm{g}} }} \frac{{\left( {\left( {ij} \right)p\left( {i,j} \right) - {\mu }_{x} {\mu }_{y} } \right)}}{{\sigma_{x} \sigma_{y} }}$	Describes linear dependency between neighboring pixels. High Correlation values indicate high local grey-level dependency, i.e. similar grey-level regions in the image
Entropy	$- \mathop \sum \limits_{i = 1}^{{N_{\rm{g}} }} \mathop \sum \limits_{j = 1}^{{N_{\rm{g}} }} p\left( {i,j} \right){ \log }\left( {p\left( {i,j} \right)} \right)$	Measure of the randomness of the texture or intensity distribution. It is (approximately) inversely correlated to the uniformity
Cluster shade	$\mathop \sum \limits_{i = 1}^{{N_{\rm{g}} }} \mathop \sum \limits_{j = 1}^{{N_{\rm{g}} }} \left( {i + j - {\mu }_{x} - {\mu }_{y} } \right)^{3} p\left( {i,j} \right)$	Measure of the skewness of the GLCM matrix. High Cluster shade value means that image is asymmetric

^a^In all equations *µ*_*x*_, *µ*_*y*_ and *σ*_*x*_, *σ*_*y*_ denote the mean and standard deviation of the row and column sums of the GLCM, respectively

The equation for the image entropy of the histogram based parameters is the same as for the GLCM parameters is described in Table 2. In the case of image entropy the *p*(*i,j*) is replaced by normalized histogram counts. Thus, as GLCM entropy describes randomness in texture (within the window limited by the displacement values), image entropy describes randomness of the grey-level values.

After *µ*CT and CBCT imaging, the osteochondral cores were subjected to histological analyses. Histological sections (3 *µ*m thick) were stained with Safranin-O and imaged with a digital pathology slide scanner (×40 magnification and 0.25 *µ*m pixel size; Aperio AT2, Leica Biosystems, Wetzlar, Germany). Subsequently, severity of osteoarthritis was evaluated using the OARSI histopathological grading system^27^ from three consecutive sections by two independent graders. In the case of disagreements, a consensus grade was used for each sample.

Pearson’s correlation coefficients were calculated to evaluate the association of morphometric parameters calculated from *µ*CT with individual histogram and GLCM texture parameters calculated from CBCT. As the OARSI grade is ordinal, correlations between the morphometric, GLCM and histogram parameters were evaluated using the Spearman’s rank correlation coefficient. Furthermore, we tested whether multiple histogram and GLCM parameters could estimate trabecular structure better compared to individual histogram or GLCM parameters by using stepwise linear regression with morphometric parameters as dependent variables (separate models) and all histogram and GLCM parameters as independent variables. Multicollinearity in stepwise linear regression was taken into account by calculating the variance inflation factor (VIF) and excluding parameters with VIF > 5^29^ from the models. Furthermore, homoscedasticity of the data and residual distribution were qualitatively evaluated from the P–P-plot of regression standardized residuals and from the residuals vs. fitted plot. These statistical tests for comparing structural information with GLCM, histogram parameters and OARSI scores were conducted for all the 53 samples (osteochondral cores).

To evaluate whether the location of core extraction affects the results, in addition to correlation coefficients for the whole dataset, the correlation coefficients for subgroups based on the origin (TKA patients and cadavers), compartmental location (medial tibial plateau and lateral tibial plateau, femora excluded), and areal location (central tibial plateau, anterior tibial plateau, posterior tibial plateau, and distal tibial plateau) are also reported (see Table 1 for subgroup sample sizes). The dependency of the location was tested also with the models generated in stepwise linear regression described above. The effect of core location was evaluated by including the subgroup information to the model to see whether it would have an effect on the correlation (change in adjusted *R*^*2*^). The number of cores per subgroup is shown in Table 1. All the statistical tests were performed with IBM SPSS Statistics software (v.25, IBM Corp., Armonk, NY, USA).

## Results

**Figure 3:**
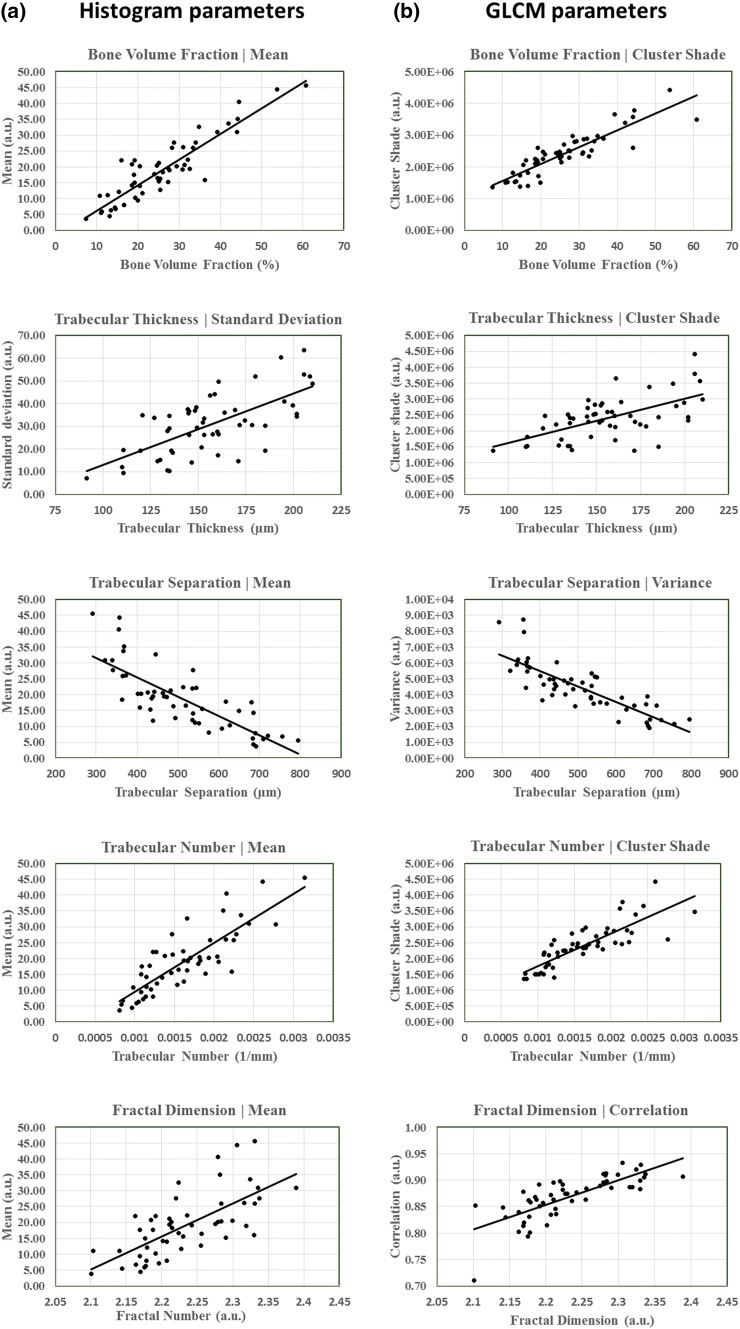
Scatter plots of the highest correlations with the histogram/GLCM parameters with the different trabecular bone morphometrics. Correlation coefficients for all parameters, including the ones presented in this figure, are presented in Table 3. (a) Scatter plots of the highest histogram parameter correlations with (from top to bottom) BV/TV, TbTh., Tb.Sp., Tb.N., and FD. (b) Scatter plots of the highest GLCM parameter correlations with (from top to bottom) BV/TV, TbTh., Tb.Sp., Tb.N., and FD.

BV/TV from *µ*CT had strong correlation (|*r*| > 0.7) with the histogram parameters (mean, standard deviation), and GLCM texture parameters (correlation, cluster shade, and variance) from CBCT (Table 3 and Fig. 3). Because BV/TV is highly associated with Tb.Th., Tb.Sp., Tb.N. and FD, it is not surprising that GLCM and histogram parameters had similar associations with these other morphometric parameters too. However, only few histogram (mean and standard deviation) and GLCM parameters (correlation and cluster shade) had strong or moderate correlations (|*r*| > 0.5) with all the morphometric parameters. BV/TV and Tb.N. had the highest correlations with mean (*r***=** 0.907 and *r* = 0.834, respectively), Tb.Th. with standard deviation (*r* = 0.676), Tb.Sp. with GLCM variance (*r* = − 0.803), and FD with GLCM correlation (*r* = 0.759). Regarding associations with OA severity, OARSI grade had moderate correlations with mean (*r* > 0.612), standard deviation (*r* > 0.663), and with GLCM cluster shade (*r* > 0.612).

**Table 3:** Correlation coefficients from comparisons between *µ*CT morphometry, CBCT GLCM and histogram parameters, and OARSI grade (*n* = 53).

	Trabecular bone morphometrics from *µ*CT				
	BV/TV (Pearson’s *R*)	Tb.Th. (Pearson’s *R*)	Tb.Sp. (Pearson’s *R*)	Tb.N. (Pearson’s *R*)	FD (Pearson’s *R*)
Histogram parameters					
Mean	0.907**	0.606**	− 0.792**	0.834**	0.691**
Standard deviation	0.891**	0.676**	− 0.757**	0.795**	0.672**
Skewness	0.060	0.191	0.083	− 0.052	− 0.097
Kurtosis	0.154	0.265	0.026	0.026	− 0.026
Image entropy	− 0.129	0.165	0.278*	− 0.215	− 0.063
GLCM texture parameters					
Contrast	− 0.059	− 0.121	− 0.104	− 0.052	− 0.185
Correlation	0.782**	0.522**	− 0.751**	0.776**	0.759**
Cluster shade	0.891**	0.600**	− 0.803**	0.825**	0.718**
ASM	− 0.346*	− 0.079	0.592**	− 0.404**	− 0.216
Entropy	0.396**	0.104	− 0.628**	0.454**	0.264
IDM	− 0.317*	− 0.035	0.571**	− 0.382**	− 0.178
Variance	0.825**	0.436**	− 0.820**	0.804**	0.640**
Histology					
OARSI (Spearman’s *ρ*)	0.584**	0.573**	− 0.461**	0.479**	0.388**

Asterisks (*) indicate for the statistical significance of the correlations (***p < 0.001, **p < 0.01, *p < 0.05). Strong correlations bolded (|*r*| > 0.7)

Subgrouping revealed that the histogram mean, GLCM cluster shade, GLCM variance and GLCM correlation had strong correlation (|*r*| > 0.7) with BV/TV both when the subgroups were considered separately (Supplementary Tables 2–5) or pooled together. Since BV/TV has dependency with other morphometrics, we conducted the stepwise linear regression only with BV/TV.

Stepwise linear regression (Table 4) revealed that the model with mean and GLCM IDM parameters yielded highest coefficient of determination (*adjusted R*^*2*^ = 0.864) with BV/TV, and when compared with the best individual parameter histogram mean the adjusted *R*^2^ was lower (adjusted *R*^2^ = 0.818, adjusted *R*^2^ change = 0.046). The model was also robust to the core extraction location as the addition of location did not improve the adjusted *R*^2^ values (TKA/Cadaver grouping: adjusted *R*^*2*^ change = − 0.001; Compartmental grouping: adjusted *R*^2^ change = − 0.002; Areal grouping: adjusted *R*^*2*^ change = − 0.001).

**Table 4:** Results and coefficient info from stepwise linear regression.

Added subgrouping	Model	Adjusted *R*^2^	Change in adjusted *R*^2^	Predictor	*B*	*ß*	*p* value	VIF
Sample origin	1	0.818	0.818	Mean	1.018	0.907	< 0.0001	1.000
	2	0.864	0.046	Mean	1.179	1.050	< 0.0001	1.435
				IDM	29.004	0.261	< 0.0001	1.435
	3	0.863	− 0.001	Mean	1.199	1.068	< 0.0001	1.628
				IDM	28.141	0.253	< 0.0001	1.471
				Subgroup	− 1.149	− 0.047	0.432	1.322
Compartmental location^a^	1	0.795	0.795	Mean	0.956	0.894	< 0.0001	1.000
	2	0.874	0.079	Mean	1.161	1.086	< 0.0001	1.457
				IDM	33.692	0.343	< 0.0001	1.457
	3	0.872	− 0.002	Mean	1.166	1.090	< 0.0001	1.552
				IDM	34.315	0.349	< 0.0001	1.667
				Subgroup	0.294	0.015	0.797	1.147
Areal location^a^	1	0.795	0.795	Mean	0.956	0.894	< 0.0001	1.000
	2	0.874	0.079	Mean	1.161	1.086	< 0.0001	1.457
				IDM	33.692	0.343	< 0.0001	1.457
	3	0.873	− 0.001	Mean	1.177	1.101	< 0.0001	1.578
				IDM	32.947	0.336	< 0.0001	1.488
				Subgroup	0.521	0.048	0.419	1.220

Models predicting BV/TV are based on stepwise linear regression, the models are generated automatically, starting with best individual histogram/GLCM parameter as predictor (histogram mean = model 1) then further “significant” predictors are added if they improve the prediction and if their variance inflation factor (VIF) is less than 5 (histogram mean + GLCM IDM = model 2). The locational dependency was evaluated by manually adding subgroup information to the models (see groups in text and Table 1), which corresponds to the model 3. Unstandardized coefficients (*B*), standardized coefficients (*ß*), and statistical significance (*p* value) of the predictors for each model are also reported in the table

All trabecular morphometric parameters had significant associations with the OARSI grade (*p* < 0.01), but the strength of the correlations varied: BV/TV and Tb.Th. had moderate correlations (*r* = 0.584 and *r* = 0.573, respectively), while Tb.Sp., Tb.N. and FD had weaker associations (*r* = − 0.461, *r* = 0.479, and *r* = 0.388, respectively) with the OARSI grade (Table 3).

With regard to subgrouping of the samples for cadavers and TKA patients, all the trabecular morphometric parameters did have weak to moderate correlations with the OARSI grade in the cadaver and TKA groups (Supplementary Table 3). From the morphometric parameters, in both groups, BV/TV yielded the highest correlation with the OARSI grade (cadaver group: *r* = 0.418, TKA group: *r* = 0.650). Subgrouping to medial and lateral tibial plateau groups (Supplementary Table 4), revealed that all the trabecular morphometric parameters in medial tibial plateau group had significant correlations ranging from weak to moderate (0.432 < |*r*| < 0.684) with the OARSI grade. BV/TV and Tb.Th. (*r* = 0.681 and *r* = 0.684, respectively) had the highest correlations with the OARSI grade in the medial subgroup. However, in the lateral tibial plateau only weak correlations of Tb.Sp. and Tb.N. with the OARSI grade were observed (*r* = − 0.462 and 0.408, respectively). Compartmental subgrouping (Supplementary Table 5) showed no significant correlations between the trabecular bone morphometrics and the OARSI grade in distal and anterior groups. However, in central tibial plateau group, the BV/TV had moderate and significant correlation with the OARSI grade (*r* = 0.673), and in posterior tibial plateau group BV/TV, Tb.Th., and Tb.Sp. had similarly significant and moderate correlations with the OARSI grade (*r* = 0.655, *r* = 0.658, and *r* = − 0.598, respectively).

## Discussion

In this study, we aimed to quantify sub-resolution subchondral bone morphometrics related to OA from clinical CBCT *ex vivo*. Our results demonstrate that sub-resolution features can be quantified, from clinical CBCT using 3D texture analysis by calculating histogram and GLCM parameters.

The primary finding of our study is that select parameters from histogram and GLCM analyses are associated with specific, micro-level morphometric structures of subchondral bone better than the others. As seen in Table 3 and Fig. 3, histogram mean, histogram standard deviation, GLCM cluster shade, GLCM correlation and GLCM variance seem to associate well with the various morphometric structures. However, our results also indicate that using only individual histogram or GLCM parameters to predict individual morphometric structures might not feasible. For example, BV/TV had strong correlation with all of the aforementioned histogram and GLCM parameters (*r* > 0.782). Even though histogram mean had the highest correlation with the BV/TV when all the cores were included, in some subgroups the strongest association was with other histogram/GLCM parameters (*e.g.* standard deviation in cadavers, medial tibial, anterior tibial, and posterior tibial plateau subgroups, and GLCM cluster shade in lateral tibial and distal tibial plateau subgroups). Since the relation of the histogram/GLCM parameters with morphometric parameters seem to vary depending on the subgrouping, the use of multiple histogram/GLCM parameters combined to predict the trabecular bone morphometrics is the most preferable option in the future.

The stepwise linear regression model combining histogram and GLCM parameters (histogram mean and GLCM IDM) was robust for the location (with all subgrouping criterias: adjusted *R*^2^ change < 0) and predicted BV/TV better when compared to the individual parameters (histogram mean: adjusted *R*^2^ = 0.818; histogram mean and IDM: adjusted *R*^2^ = 0.864). As the correlation between individual histogram mean and BV/TV is already strong, only a slight increase in coefficient of determination (adjusted *R*^2^ change = 0.047) of combined histogram mean and GLCM IDM with BV/TV might seem redundant. However, it should be noted that this study was conducted with small *ex vivo* osteochondral cores. This set limitations as the imaging and reconstruction protocols are optimized for *in vivo* limbs. First of all, we are lacking the noise generated from the soft tissue and surrounding bone matrix which is present *in vivo.* Secondly, this study uses only 8-bit images as the imaging and reconstruction parameters did limit the full utilization of the 16-bit dynamic range for small osteochondral cores. The combination of the first and second order grey-level statistics could be advantageous in reducing the effect of noise in the analysis of the *in vivo* clinical CT data. This is true especially if 16-bit dynamic range can be utilized.

In this study, the used voxel size was 200 *µ*m. Due to the technical advancements in current CBCT systems, the high resolution scans of extremities with minimal radiation dose has been surfaced to clinical practice.^25^ For example, the CBCT scanner used in this study has been reported to cause only 6 *µ*Sv effective dose (with 200 *µ*m resolution) in a CBCT scan of the ankle, which compares to the effective dose of 4 conventional 2D radiographs from the same anatomical region.^18^ The histogram/GLCM analysis might be best suited for clinical CBCT images of extremities, especially because the multidetector CT (MDCT) systems provide poorer resolution (~ 0.6mm voxel size) with higher radiation dose.

Other approaches determining trabecular bone morphometrics from clinical resolution images have been proposed. Methods utilizing peripheral quantitative CT and *µ*MRI can reach resolutions around 80–140 *µ*m voxel size, and thus, direct estimates of bone strength could be calculated.^3^,^28^ However, with those methods, sufficient resolution is required as they often require binarization steps. In our study, the resolution of CBCT 200 *µ*m was not sufficient for binarization as the average trabecular structure thickness varied from 0.5–1 voxel side lengths. In this type of scenario, the partial volume effect prevents the differentiation of trabecular bone from the trabecular cavities and the conventional selection of the threshold values based on the known attenuation values of bone is not possible. Approaches using fuzzy skeletonization can partially overcome the problems of binarization and these methods have proven good results from MDCT scans with clinical resolution images (~ 200 *µ*m voxel size).^6^ Unfortunately, Chen et al. reported that the accuracy of this method decreases with VOI diameter smaller than 5.25 mm,^6^ and in our study the core diameter restricted the VOI diameter to 3 mm. Therefore, comparison of those methods with GLCM/histogram analysis remains to be elucidated in future studies with larger samples.

The combined histogram/GLCM parameters yielded better association with the morphometrics than the individual histogram/GLCM parameters. As this shows promise for future studies, one must be aware of collinearity and autocorrelation when creating these types of multiparameter models from GLCM/histogram parameters. There are mathematical based collinearities and autocorrelations between the parameters, e.g. between mean and standard deviation where both are dependent on the grey-level average. Another example includes inverse relationships between some of the GLCM parameters, such as GLCM IDM and GLCM contrast, i.e., another value increases the other value tends to decrease. In this study we considered both positive and negative relations as an advantage in interpretation of our results. Often with multiple correlations, the simultaneous statistical interference might result in Type I errors, and consequently, *p* values require correction. Here the interpretation of the individual significant correlations of the histogram and GLCM parameters were always compared with the correlations of the variable with dependency (*i.e.* previously mentioned mean and standard deviation) before making conclusions about the significance of the parameter correlations. Thus, understanding of the parameters’ descriptive properties in addition to the mathematical base is crucial when generating more complex multiparameter prediction models.

The association of trabecular bone morphometric parameters with the histopathological OARSI grade were in line with our previous study.^10^ With the increasing OARSI grade, which evaluates the progressive degeneration of articular cartilage, we found a similar decrease in trabecular bone separation and increases in trabecular bone volume fraction, trabecular thickness, trabecular number and fractal dimension. The main difference compared to our previous study is that the current associations (correlation coefficients) between the trabecular bone morphometrics and the OARSI grade are only weak to moderate, whilst in the previous study the correlations were strong (*|r|* > 0.7). This difference might be related to a different and smaller patient population here. Furthermore, the associations between the OARSI grade and structural parameters varied in different subgroups, which supports strong dependence on the sample location. Thus, more controlled core extraction location-wise would be preferable in future studies when investigating these kinds of associations. Interestingly, OARSI had stronger correlations with histogram mean, histogram standard deviation and GLCM cluster shade (*r* = 0.612, *r* = 0.664, and *r* = 0.612, respectively) while the highest morphometric parameter association with the OARSI grade yielded *r* > 0.584(BV/TV). This gives indication that the underlying image texture/grey level information could be used in classification of OA severity from clinical CT bone data. These associations between the histogram/GLCM parameters related to OA severity need to be investigated with a larger patient population. Still, a smaller patient population in this study does not restrict us from making general conclusions on the associations between local trabecular bone structure and histogram/GLCM parameters calculated from clinical CT images.

In conclusion, we demonstrated that GLCM texture and histogram-based parameters from trabecular bone are associated with sub-resolution morphometrics in CBCT *ex vivo*. Our results also suggest that sub-resolution morphometrics can be predicted from clinical CBCT images even more accurately when combining histogram and GLCM texture based parameters. These methods show great potential for deriving the microstructural subchondral bone alterations from clinical CBCT. However, further studies of the feasibility of the combined use of histogram and GLCM based parameters for *in vivo* are required.

## Electronic supplementary material

Below is the link to the electronic supplementary material.

Supplementary material 1 (PDF 841 kb)
