# Supplementary material for: Quantifying Subresolution 3D Morphology of Bone with Clinical Computed Tomography
Source: Ann Biomed Eng. 2019 Oct 3;48(2):595–605. doi: 10.1007/s10439-019-02374-2 (PMC6949315; doi:10.1007/s10439-019-02374-2)

Supplementary material

All Supplementary data (Supplementary Tables 1-6, and Supplementary Figure 1) is presented in this document.

Supplementary Table 1. *Representation of the 13 unique displacements utilized in GLCM.*

**Direction (*θ*, *φ*)**	**Displacement in 3D matrix d(x, y, z)**
(- , 0°)	(0, 0, 1)
(90°, 45°)	(0, 1, 1)
(90°,90°)	(0, 1, 0)
(90°,135°)	(0, 1, -1)
(0°, 45°)	(1, 0, 1)
(0°, 90°)	(1, 0, 0)
(0°, 135°)	(1, 0, -1)
(45°, 45°)	(1, 1, 1)
(45°, 90°)	(1, 1, 0)
(45°, 135°)	(1, 1, -1)
(135°, 45°)	(-1, 1, 1)
(135°, 90°)	(-1, 1, 0)
(135°, 135°)	(-1, 1, -1)

Supplementary Table 2. *Descriptive statistics of μCT morphometrics (mean±standard deviation), CBCT histogram/GLCM parameters(mean±standard deviation), and OARSI(median and minimum to maximum range) in different subgroups. Histogram/GLCM parameters are all arbitrary values thus no units in the table*.

a. grey-level co-occurrence matrix
b. bone volume fraction
c. trabecular thickness
d. trabecular separation
e. trabecular number
f. fractal dimension

Location subgroups	Morphometrics (mean±standard deviation)					Histogram parameters (mean±standard deviation)					GLCM[a] parameters (mean±standard deviation)							OARSI (median) {min;max}
Sample Origin	BV/TV[b] (%)	Tb.Th.[c] (μm)	Tb.Sp.[d] (μm)	Tb.N.[e] (1/μm)	FD[f] (A.U.)	Mean	SD[g]	Skewness	Kurtosis	Image Entropy	Contrast	Correlation	CS[h] (x10^6)	ASM[i]	Entropy	IDM[j]	Variance (x10^3)	
**Cadavers**	23.2±8.6	150.1±22.9	553.5±130.7	1.5±0.5	2.2±0.1	16.0±7.8	26.6±10.7	3.9±0.8	21.2±8.5	0.94±0.08	773.9±135.1	0.86±0.04	2.2±0.5	0.32±0.12	4.7±1.0	0.60±0.09	4.0±1.2	2.50 {1.0;4.5}
**TKA[k] patients**	32.2±13.7	172.8±33.9	442.8±93.3	1.8±0.6	2.2±0.1	26.4±10.6	40.9±13.2	4.0±1.4	24.1±18.2	0.91±0.10	850.2±144.3	0.89±0.03	2.7±0.7	0.22±0.10	5.6±0.9	0.52±0.09	5.6±1.6	4.50 {1.0;6.5}
**Compartmental location**																		
**Medial tibial plateu**	25.6±11.5	160.3±31.8	544.7±133.6	1.6±0.6	2.2±0.1	18.2±10.3	30.2±14.3	4.2±1.2	25.0±15.4	0.96±0.06	777.0±128.3	0.87±0.05	2.4±0.7	0.32±0.09	4.7±0.8	0.61±0.07	4.2±1.4	2.75 {1.0;5.0}
**Lateral tibial plateu**	23.0±7.9	145.9±20.8	497.3±119.8	1.6±0.4	2.2±0.1	17.3±8.2	27.6±10.5	3.9±0.8	21.6±8.8	0.90±0.10	822.4±159.2	0.86±0.03	2.3±0.6	0.27±0.14	5.2±1.2	0.55±0.12	4.4±1.5	2.50 {1.0;6.5}
**Areal location**																		
**Central tibial plateu**	30.8±9.3	171.7±23.4	450.4±111.2	1.8±0.5	2.3±0.1	25.9±7.3	40.0±7.2	4.1±0.7	22.8±7.9	0.88±0.10	782.2±107.7	0.89±0.02	2.7±0.5	0.21±0.10	5.7±0.7	0.51±0.07	5.4±1.4	2.75 {1.5;6.5}
**Anterior tibial plateu**	23.7±12.2	150.5±24.3	538.4±137.3	1.5±0.6	2.2±0.1	16.7±10.4	26.5±14.8	3.9±1.5	22.4±19.7	0.94±0.09	806.1±136.0	0.86±0.04	2.3±0.8	0.31±0.12	4.8±1.1	0.59±0.11	4.3±1.6	2.50 {1.0;5.0}
**Posterior tibial plateu**	20.7±8.9	138.8±29.4	549.2±140.4	1.4±0.4	2.2±0.1	14.9±8.9	25.2±12.2	3.8±0.6	19.4±4.6	0.91±0.09	848.4±189.0	0.84±0.05	2.1±0.5	0.30±0.15	4.9±1.3	0.58±0.12	3.9±1.4	3.75 {1.0;4.5}
**Distal tibial plateu**	22.8±5.8	153.0±22.7	530.8±100.7	1.5±0.4	2.2±0.0	14.8±5.3	25.6±8.3	4.3±1.0	27.7±11.0	0.98±0.04	766.3±124.2	0.86±0.03	2.2±4.4	0.34±0.09	4.5±0.8	0.62±0.07	3.8±1.0	1.50 {1.0;4.0}

g. standard deviation
h. cluster shade
i. angular second moment
j. inverse difference moment
k. total knee arthroplasty

Supplementary Table 3. *Correlation coefficients from µCT data morphometrics vs CBCT data histogram and GLCM based parameters from the sample origin subgroups. On the right, correlation coefficients for morphometrics vs OARSI. Asterisks(*) indicate for the statistical significance of the correlations (***: p<0.001, **: p<0.01, *:p<0.05). Strong correlations bolded (|r| >0.7).*

Location		BV/TV (Pearson's R)	Tb.Th. (Pearson's R)	Tb.Sp. (Pearson's R)	Tb.N, (Pearson's R)	FD (Pearson's R)	OARSI (Spearman's ρ)	Morphometric	OARSI (Spearman's ρ)
**Cadavers n=38**	**Histogram parameters**							**BV/TV**	0.418**
	**Mean**	**0.883****	0.462**	**-0.818****	**0.836****	**0.785****	0.455**	**Tb.Th.**	0.329*
	**Standard Deviation**	**0.892****	0.592**	**-0.757****	**0.790****	**0.750****	0.492**	**Tb.Sp.**	-0.352*
	**Skewness**	-0.093	0.150	0.185	-0.201	-0.199	0.035	**Tb.N.**	0.383*
	**Kurtosis**	-0.064	0.241	0.186	-0.197	-0.175	-0.009	**FD**	0.352*
	**Image Entropy**	-0.213	0.183	0.358*	-0.313	-0.229	-0.235		
	**GLCM texture parameters**								
	**Contrast**	-0.154	-0.117	0.027	-0.159	-0.228	0.108		
	**Correlation**	**0.798****	0.387*	**-0.775****	**0.799****	**0.803****	0.281		
	**Cluster Shade**	**0.876****	0.450**	**-0.838****	**0.825****	**0.781****	0.429**		
	**Angular Second Moment**	-0.430**	-0.053	0.638**	-0.490**	-0.385*	-0.297		
	**Entropy**	0.487**	0.083	-0.684**	0.547**	0.449**	0.316		
	**Inverse Difference Moment**	-0.411*	-0.022	0.632**	-0.479**	-0.371*	-0.302		
	**Variance**	**0.785****	0.247	**-0.870****	**0.816****	**0.755****	0.424**		
								**Morphometric**	**OARSI (Spearman's ρ)**
**TKA patients n=15**	**Histogram parameters**								
	**Mean**	**0.915****	0.616*	**-0.752****	**0.863****	**0.820****	0.580*	**BV/TV**	0.650**
	**Standard Deviation**	**0.874****	0.651**	**-0.721****	**0.824****	**0.837****	0.623*	**Tb.Th.**	0.547*
	**Skewness**	0.183	0.229	-0.047	0.113	0.054	-0.190	**Tb.Sp.**	-0.448
	**Kurtosis**	0.268	0.255	-0.115	0.203	0.155	-0.155	**Tb.N.**	0.441
	**Image Entropy**	0.065	0.290	-0.014	0.018	0.284	0.117	**FD**	0.520*
	**GLCM texture parameters**								
	**Contrast**	-0.186	-0.423	-0.212	-0.029	-0.097	-0.511		
	**Correlation**	**0.859****	**0.773****	-0.501	**0.714****	**0.757****	**0.772****		
	**Cluster Shade**	**0.875****	0.617*	**-0.765****	**0.841****	**0.886****	0.506		
	**Angular Second Moment**	0.061	0.288	0.137	-0.009	0.255	0.246		
	**Entropy**	0.002	-0.268	-0.176	0.074	-0.197	-0.146		
	**Inverse Difference Moment**	0.093	0.354	0.127	0.002	0.275	0.285		
	**Variance**	**0.814****	0.430	**-0.722****	**0.792****	0.680**	0.251		

Supplementary Table 4. *Correlation coefficients from µCT data morphometrics vs CBCT data histogram and GLCM based parameters from the compartmental location subgroups. On the right, correlation coefficients for morphometrics vs OARSI. Asterisks(*) indicate for the statistical significance of the correlations (***: $p<0.001$, **: $p<0.01$, *:$p<0.05$). Strong correlations bolded ($|r| >0.7$).*

Location		BV/TV (Pearson's R)	Tb.Th. (Pearson's R)	Tb.Sp. (Pearson's R)	Tb.N, (Pearson's R)	FD (Pearson's R)	OARSI (Spearman's ρ)	Morphometric	OARSI (Spearman's ρ)
**Medial tibial plateu**	**Histogram parameters**							**BV/TV**	0.681**
	**Mean**	**0.926****	0.691**	**-0.793****	**0.796****	0.679**	0.604**	**Tb.Th.**	0.684**
**n=22**	**Standard Deviation**	**0.941****	**0.790****	**-0.777****	**0.779****	0.678**	0.670**	**Tb.Sp.**	-0.479*
	**Skewness**	0.394	0.481*	-0.063	0.161	0.023	0.526*	**Tb.N.**	0.528*
	**Kurtosis**	0.475*	0.511*	-0.160	0.249	0.099	0.518*	**FD**	0.432*
	**Image Entropy**	-0.440*	-0.141	0.443*	-0.466*	-0.416	-0.171		
	**GLCM texture parameters**								
	**Contrast**	-0.231	-0.245	0.142	-0.247	-0.354	-0.206		
	**Correlation**	**0.808****	0.626**	**-0.765****	**0.761****	**0.763****	0.538**		
	**Cluster Shade**	**0.934****	0.666**	**-0.819****	**0.812****	0.693**	0.605**		
	**Angular Second Moment**	-0.544**	-0.379	0.536*	-0.501*	-0.437*	-0.245		
	**Entropy**	0.597**	0.418	-0.600**	0.555**	0.493*	0.292		
	**Inverse Difference Moment**	-0.532*	-0.360	0.527*	-0.487*	-0.412	-0.269		
	**Variance**	**0.870****	0.532*	**-0.821****	**0.798****	0.675**	0.408		
								**Morphometric**	**OARSI (Spearman's ρ)**
**Lateral tibial plateu**	**Histogram parameters**								
	**Mean**	**0.855****	0.429*	**-0.797****	**0.820****	0.655**	0.566**	**BV/TV**	0.375
**n=24**	**Standard Deviation**	**0.825****	0.442*	**-0.769****	**0.798****	0.638**	0.621**	**Tb.Th.**	0.312
	**Skewness**	-0.195	-0.133	0.085	-0.176	-0.184	-0.326	**Tb.Sp.**	-0.462*
	**Kurtosis**	-0.083	0.005	0.084	-0.118	-0.118	-0.258	**Tb.N.**	0.408*
	**Image Entropy**	0.197	0.458*	0.105	0.051	0.177	-0.015	**FD**	0.265
	**GLCM texture parameters**								
	**Contrast**	0.129	0.131	-0.274	0.077	-0.136	0.506*		
	**Correlation**	**0.765****	0.198	**-0.745****	**0.832****	**0.781****	0.183		
	**Cluster Shade**	**0.876****	0.450*	**-0.829****	**0.840****	0.695**	0.569**		
	**Angular Second Moment**	-0.237	0.178	0.661**	-0.372	-0.091	-0.242		
	**Entropy**	0.267	-0.167	-0.667**	0.399	0.122	0.261		
	**Inverse Difference Moment**	-0.191	0.227	0.628**	-0.335	-0.044	-0.249		
	**Variance**	**0.770****	0.325	**-0.798****	**0.742****	0.541**	0.508*		

Supplementary Table 5. *Correlation coefficients from µCT data morphometrics vs CBCT data histogram and GLCM based parameters from the areal location subgroups. On the right, correlation coefficients for morphometrics vs OARSI. Asterisks(*) indicate for the statistical significance of the correlations (***: p<0.001, **: p<0.01, *:p<0.05).Strong correlations bolded (|r| >0.7).*

Location		BV/TV (Pearson's R)	Tb.Th. (Pearson's R)	Tb.Sp. (Pearson's R)	Tb.N, (Pearson's R)	FD (Pearson's R)	OARSI (Spearman's ρ)	Morphometric	OARSI (Spearman's ρ)
Central tibial plateu	Histogram parameters							BV/TV	0.673*
	Mean	**0.909****	0.244	**-0.760***	**0.768****	0.689*	0.567	Tb.Th.	0.424
n=10	Standard Deviation	**0.898****	0.380	-0.638*	0.694*	0.606	0.517	Tb.Sp.	-0.605
	Skewness	-0.226	0.502	0.522	-0.447	-0.491	-0.075	Tb.N.	0.492
	Kurtosis	0.008	**0.728***	0.404	-0.324	-0.396	0.218	FD	0.362
	Image Entropy	0.076	**0.829****	0.519	-0.259	-0.223	0.081		
	GLCM texture parameters								
	Contrast	0.090	0.271	-0.113	-0.099	-0.206	-0.131		
	Correlation	**0.839****	0.096	**-0.731***	**0.805****	**0.789****	0.692*		
	Cluster Shade	**0.767****	0.422	-0.566	0.539	0.454	0.698*		
	Angular Second Moment	-0.054	**0.767****	0.651*	-0.352	-0.310	0.181		
	Entropy	0.133	**-0.727***	**-0.712***	0.412	0.366	-0.081		
	Inverse Difference Moment	-0.047	**0.745***	0.647*	-0.334	-0.291	0.081		
	Variance	**0.752***	0.201	**-0.721***	0.618	0.527	0.692*		
								Morphometric	OARSI (Spearman's ρ)
Anterior tibial plateu	Histogram parameters								
	Mean	**0.859****	0.543	**-0.755****	**0.833****	0.620*	0.281	BV/TV	0.502
n=11	Standard Deviation	**0.929****	0.642*	**-0.773****	**0.903****	**0.742****	0.544	Tb.Th.	0.590
	Skewness	0.646*	**0.740****	-0.185	0.433	0.251	0.244	Tb.Sp.	-0.373
	Kurtosis	**0.767****	**0.784****	-0.336	0.571	0.364	0.263	Tb.N.	0.433
	Image Entropy	0.368	0.626*	-0.021	0.305	0.543	0.157	FD	0.493
	GLCM texture parameters								
	Contrast	-0.246	-0.383	-0.107	-0.194	-0.357	0.267		
	Correlation	**0.825****	0.376	**-0.835****	**0.899****	**0.790****	0.005		
	Cluster Shade	**0.970****	0.657*	**-0.820****	**0.950****	**0.802****	0.461		
	Angular Second Moment	0.119	0.365	0.202	0.079	0.361	0.226		
	Entropy	-0.104	-0.391	-0.238	-0.048	-0.332	-0.203		
	Inverse Difference Moment	0.156	0.408	0.173	0.113	0.399	0.203		
	Variance	**0.701***	0.270	-0.787**	**0.718***	0.439	0.134		
								Morphometric	OARSI (Spearman's ρ)
Posterior tibial plateu	Histogram parameters								
	Mean	**0.943****	**0.780****	**-0.822****	**0.846****	**0.729****	**0.766****	BV/TV	0.655*
n=12	Standard Deviation	**0.960****	**0.857****	**-0.819****	**0.836****	**0.714****	**0.777****	Tb.Th.	0.658*
	Skewness	0.008	-0.028	-0.025	-0.013	-0.014	0.161	Tb.Sp.	-0.598*
	Kurtosis	-0.060	-0.007	0.126	-0.093	-0.004	0.233	Tb.N.	0.495
	Image Entropy	-0.444	-0.256	0.337	-0.454	-0.434	-0.157	FD	0.505
	GLCM texture parameters								
	Contrast	-0.102	-0.074	-0.025	-0.149	-0.309	0.304		
	Correlation	**0.781****	0.631*	-0.664*	**0.769****	**0.784****	0.422		
	Cluster Shade	**0.916****	**0.759****	**-0.867****	**0.831****	0.675*	**0.766****		
	Angular Second Moment	-0.540	-0.289	**0.726****	-0.571	-0.354	-0.648*		
	Entropy	0.640*	0.388	**-0.777****	0.656*	0.454	0.669*		
	Inverse Difference Moment	-0.572	-0.312	**0.751****	-0.600*	-0.371	-0.648*		
	Variance	**0.856****	0.612*	**-0.829****	**0.823****	0.693*	**0.787****		
								Morphometric	OARSI (Spearman's ρ)
Distal tibial plateu	Histogram parameters								
	Mean	**0.720****	0.269	-0.637*	**0.736****	0.670*	0.559*	BV/TV	0.265
n=13	Standard Deviation	0.599*	0.357	-0.533	0.599*	0.516	0.652*	Tb.Th.	0.303
	Skewness	-0.374	-0.258	0.181	-0.346	-0.352	0.265	Tb.Sp.	-0.061
	Kurtosis	-0.258	-0.104	0.115	-0.280	-0.297	0.297	Tb.N.	0.146
	Image Entropy	0.401	0.549	-0.060	0.170	0.352	-0.125	FD	0.169
	GLCM texture parameters								
	Contrast	0.374	0.110	-0.412	0.368	0.225	0.006		
	Correlation	**0.742****	-0.016	-0.659*	**0.808****	**0.907****	-0.064		
	Cluster Shade	**0.846****	-0.005	**-0.791****	**0.846****	**0.824****	0.344		
	Angular Second Moment	-0.478	0.324	**0.874****	**-0.780****	-0.621*	0.023		
	Entropy	0.555*	-0.324	**-0.901****	**0.835****	0.681*	0.032		
	Inverse Difference Moment	-0.467	0.280	**0.841****	**-0.747****	-0.582*	-0.006		
	Variance	**0.797****	-0.022	**-0.802****	**0.846****	**0.764****	0.195		

Supplementary Table 6. *User modifiable reconstruction parameters for the µCT and CBCT scans used in this study. Refer to Nrecon User manual and Planmed Verity User manual for further details of the µCT and CBCT parameters, respectively.*

CT system	Reconstruction parameter	Value
**µCT Skyscan 1272**	Reconstruction Angular Range	360 deg
	Smoothing	3
	Smoothing kernel	2 (Gaussian)
	Ring Artifact correction	10
	Beam Hardening Correction	30%
	Minimum for dynamic range window	0.00
	Maximum for dynamic range window	0.13
**CBCT Planmed Verity**	Reconstruction Filter(kernel)	Standard (default)
	Noise Filter	None
	Metal suppression	Off

Supplementary Figure 1. *Co-registered figure showing comparison of μCT and CBCT derived trabecular bone structure. Scale line (1 mm) presented at the bottom right of the figure.*

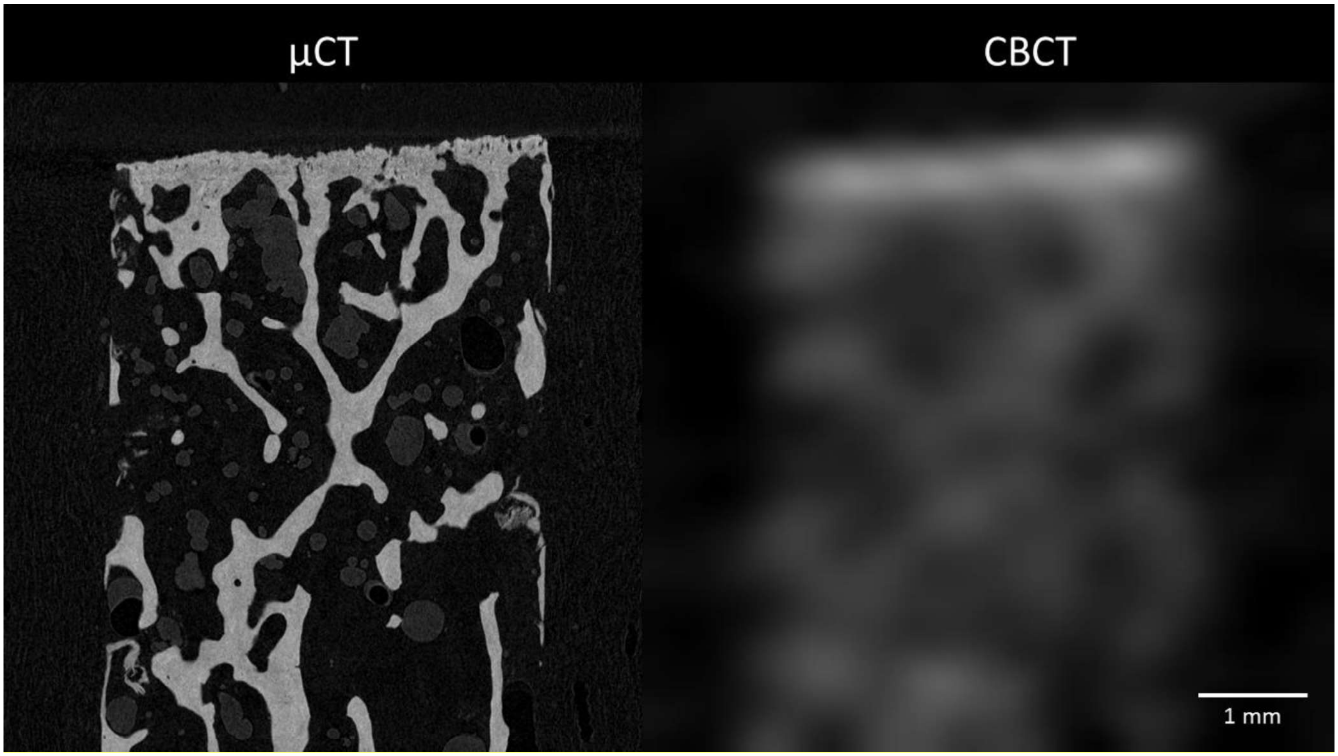

Supplement: Supplementary file 1 — Supplementary material 1 (PDF 841 kb) [file 10439_2019_2374_MOESM1_ESM.pdf]
